# New acylides: synthesis of 3-*O*-[γ-(4-oxo-2-aryl-thiazolidin-3-yl)butyryl]erythromycin A derivatives

**DOI:** 10.3762/bjoc.4.14

**Published:** 2008-05-13

**Authors:** Deepa Pandey, Wahajul Haq, Seturam B Katti

**Affiliations:** 1 Medicinal and Process Chemistry Division, Central Drug Research Institute, Lucknow, 226 001 India. Telephone: +91 0522 2612411-18 ext. 4364; fax: +91 0522 2623405

## Abstract

In search of new erythromycin derivatives 3-*O*-[γ-(4-oxo-2-aryl-thiazolidin-3-yl)butyryl]erythromycin A derivatives have been synthesized. The 3-hydroxy group was derivatised to a primary amine and subsequently the thiazolidinone nucleus was generated at the amino functionality through DCC mediated one-pot three-component reaction in good yields.

## Background

**Figure 1:**
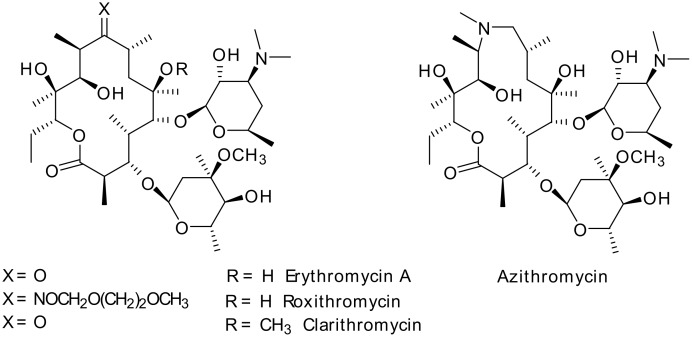
Second generation macrolides.

**Figure 2:**
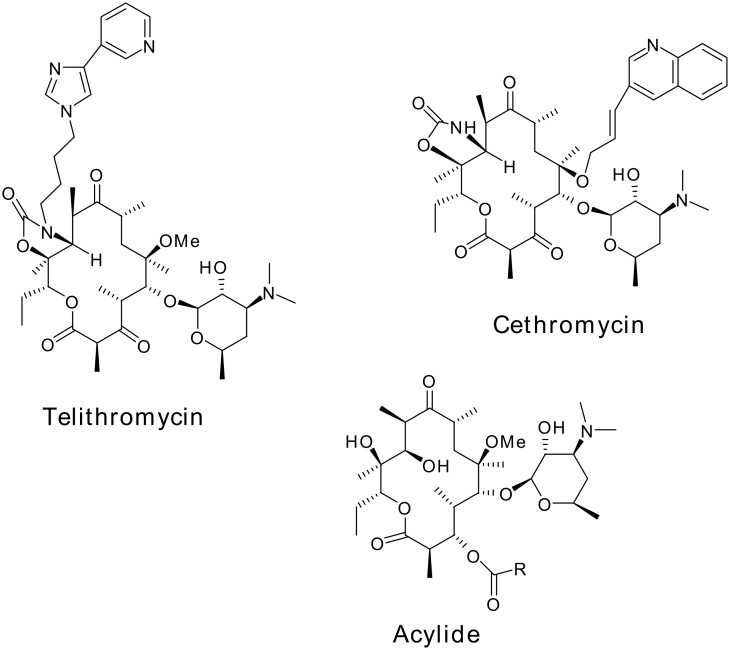
Ketolides and acylides.

Second-generation macrolides, namely clarithromycin (CAM), roxithromycin and azithromycin (Figure 1), provide good coverage against all key respiratory pathogens [1–4]. In spite of their better activity, the development of acquired resistance remains unabated. Structural modifications in the macrolides have been the most important approach for the development of novel antibacterials active against resistant strains of bacteria. Further structural modifications on decladinosylmacrolides have resulted in the identification of ketolides such as telithromycin (Aventis) [5] and cethromycin (Abbott) [6–7], and acylide (3-*O*-acyl derivatives of decladinosyl-6-*O*-methylerythromycin) derivatives [8–9] (Figure 2).

However structural modification and generation of new prototypes has been challenging due to structural complexity of the erythromycin molecule. Therefore, development of new strategies for the synthesis of novel structures is of prime importance in the area of macrolides.

Acylides are a promising new class of macrolide antibiotics [8–9]. These derivatives are active against erythromycin resistant strains and the activity is comparable to ketolides such as telithromycin. It is important to note that 3-*O*-acyl derivatives with nitrogen heterocyclic moieties, namely pyridylacetyl, were mostly active [9]. This encouraged us to explore the synthesis of 3-*O*-acylides bearing other heterocyclic systems for example thiazolidinone in place of the pyridyl residue. The thiazolidinone functionality in the molecule may be advantageous for antibacterial activity in view of similar system having been reported as antibacterials [10–11]. The thiazolidin-4-ones can be generated under very mild conditions using the DCC mediated one-pot synthesis reported by us [12].

## Results and Discussion

Decladinosyl-6-*O*-methylerythromycin A (**1**) was generated using the method available in the literature [13]. We have developed a synthesis where the 3-OH group of **1** was functionalized to an amino group using the γ-aminobutyryl spacer and subsequently a variety of thiazolidinone moieties were generated at the amino group. We have explored other spacers for generating the amino group e.g. Z-Gly, Z-Ala and Z-β-Ala etc. but due to severe steric hindrance the formation of the thiazolidinone was not successful. Therefore we have utilized the γ-aminobutyric acid (γ-Abu) as spacer for the present synthesis.

**Scheme 1:**
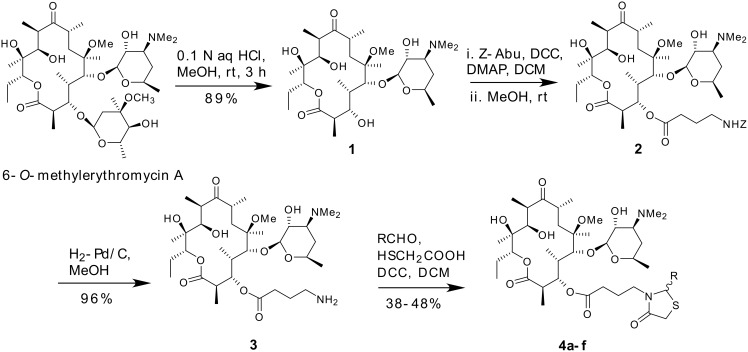
Synthesis of 3-*O*-[γ-(4-oxo-2-aryl-thiazolidin-3-yl)butyryl]-6-*O*-methylerythromycin A derivatives **4a**–**f** (Method A); R = **4a**: phenyl, **4b**: 4-chlorophenyl, **4c**: 4-fluorophenyl, **4d**: 4-methoxyphenyl, **4e**: 4-nitrophenyl and **4f**: 4-quinolyl.

Starting from clarithromycin the novel derivatives **4a**–**f** have been synthesized as shown in Scheme 1. Reaction of **1** with γ-[(Benzyloxycarbonyl)amino]butyric acid using diisopropylcarbodiimide mediated coupling in the presence of 4-(dimethylamino)pyridine resulted in the formation of a 2’,3-disubstituted acylide intermediate which upon treatment with methanol for several hours gave the desired 3-*O*-acyl derivative **2** in 72% yield after silica gel column chromatography. Compound **2** was subjected to catalytic hydrogenation using 10% Pd/C in methanol. The reaction was complete within 1 h as monitored by tlc. After usual work up the desired γ-aminoacylide derivative **3** was obtained in ~96% yield. The amino group (of compound **3**) thus obtained was utilized to append the desired thiazolidinone described below. The desired thiazolidinone was generated on **3** by dicyclohexylcarbodiimide-mediated three-component one-pot reaction, in which the amine **3** was reacted with appropriate aldehydes and mercaptoacetic acid (1:4:6 molar equivalents) in dichloromethane followed by addition of DCC at room temperature. The reaction was completed within 3–4 h as monitored by tlc. After the usual work up the desired 3-*O*-[γ-(4-oxo-2-aryl-thiazolidin-3-yl)butyryl]-6-*O*-methylerythromycin A derivatives **4a**–**f** were obtained in 38–48% isolated yields.

**Scheme 2:**
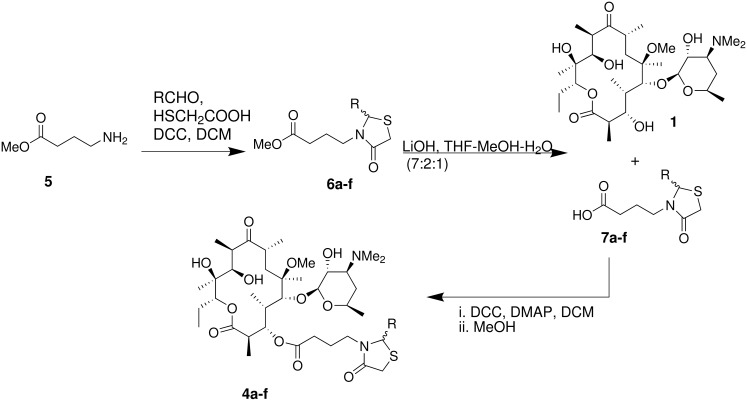
Synthesis of 3-*O*-[γ-(4-oxo-2-aryl-thiazolidin-3-yl)butyryl]erythromycin A derivatives **4a**–**f** (Method B).

An alternate approach for the synthesis of compounds **4a**–**f** was also attempted (Scheme 2). γ-Aminobutyric acid methyl ester (**5**) was treated with appropriate aldehydes in presence of mercaptoacetic acid and DCC to generate methyl γ-(4-oxo-2-aryl-thiazolidin-3-yl)butyrates **6a**–**f**. The methyl ester was subjected to alkaline hydrolysis to generate γ-(4-oxo-2-aryl-thiazolidin-3-yl)butyric acids **7a**–**f**. The thiazolidinyl acids **7a**–**f** were treated with 3-hydroxy derivative **1** in presence of DCC and DMAP to furnish the desired 3-*O*-[γ-(4-oxo-2-aryl-thiazolidin-3-yl)butyryl]erythromycin A derivatives **4a**–**f**. This reaction resulted in very low yield (~5–10%) of the desired compounds and most of the unreacted starting material was recovered. This may be attributed to the steric hindrance at 3-OH of **1** and also the large size of γ-(4-oxo-2-aryl-thiazolidin-3-yl)butyric acids **7a**–**f** compared to Z-Abu, which couples readily with the 3-OH of **1** in the same reaction conditions. We have also observed similar problems while coupling 3-*O*-decladinosyl-5-*O*-desosaminylerythronolide A (**1**) with Z-Ala and Z-Phe at 3-OH. Therefore we conclude that method A is superior to method B for the synthesis of 3-*O*-[γ-(4-oxo-2-aryl-thiazolidin-3-yl)butyryl]-6-*O*-methylerythromycin A derivatives **4a**–**f**.

## Conclusion

In summary, a facile synthesis of a novel series of substituted 3-*O*-acylides has been developed. Synthesis of the thiazolidinone moiety on the amino funtionalized erythromycin derivative has been found to be better as compared to the attachment of thiazolidinone bearing carboxylic acids at 3-OH of the erythromycin derivative. The present procedure offers a straightforward synthetic approach with minimal protection for the synthesis of a variety of derivatives in moderate yields. The mild experimental conditions are very much suitable for the highly sensitive macrolide molecule.

## Experimental

Refer to Supporting Information File 1 for full experimental data.

## Supporting Information

File 1 This file describes the full experimental details and characterization data of compounds **1**–**3**, **4a**–**f, 6a** and **7a**.

File 2 ^1^H NMR spectra of compounds **4a**–**d**.

File 3 ^13^C NMR spectra of compounds **4a**–**b** and **4d**–**e**.
