# Supplementary material for: New acylides: synthesis of 3-O-[γ-(4-oxo-2-aryl-thiazolidin-3-yl)butyryl]erythromycin A derivatives
Source: Beilstein J Org Chem. 2008 May 13;4:14. doi: 10.3762/bjoc.4.14 (PMC2486485; doi:10.3762/bjoc.4.14)
Supplement: File 1 — This file describes the full experimental details and characterization data of compounds 1–3, 4a–f, 6a and 7a. [file Beilstein_J_Org_Chem-04-14-s001.doc]

**New acylides: synthesis of 3-*O*-[γ-(4-oxo-2-aryl-thiazolidin-3-yl)butyryl]erythromycin A derivatives**

Deepa Pandey, Wahajul Haq* and Seturam B. Katti

Medicinal and Process Chemistry Division, Central Drug Research Institute, Lucknow, 226 001 India

Haq_w59@yahoo.com

Contents

i. Experimental Section 2

ii. Experimental details and characterization of **1**. 2–3

iii. Experimental details and characterization of **2** 3–4

iv. Experimental details and characterization of **3** 4–5

v. Experimental details and characterization of **4a** (method A) 5–6

vi. Experimental details and characterization of **4b** (method A) 6–7

vii. Experimental details and characterization of **4c** (method A) 7–8

viii. Experimental details and characterization of **4d** (method A) 8–9

ix. Experimental details and characterization of **4e** (method A) 9–10

x. Experimental details and characterization of **4f** (method A) 11–12

xi. Experimental details and characterization of **6a** 12

xii. Experimental details and characterization of **7a** 12–13

xiii. Experimental details and characterization of **4a** (method B) 13–14

xiv. ^1^H NMR spectra: **4a, 4b, 4c, 4d** (separate file) 15–18

xv. ^13^C NMR spectra: **4a, 4b, 4d, 4e** (separate file) 19–22

# Supporting Information

## Experimental Section

All reagents and solvents were purchased commercially and used without purification unless otherwise noted. ^1^H and ^13^C nuclear magnetic resonance (NMR) spectra were recorded in CDCl_3_ on a Bruker DRX-400 or DRX-300 FT-NMR spectrometer. Chemical shifts are reported in part per million (ppm) with tetramethylsilane (TMS) as an internal standard. Coupling constants (J) are given in hertz (Hz) and the abbreviations s, d, t, q, br and m refer to singlet, doublet, triplet, quartet, broad and multiplet respectively. Mass spectra (MS) were obtained with a JEOL-SX-102 instrument using fast atom bombardment (FAB positive) technique. Infrared spectra (IR) were recorded on a Perkin-Elmer FT-IR spectrometer as KBr pellets and are reported as reciprocal centimeters (cm^−1^). The C, H, N analyses were performed on CARLO-ERBA EA1108 elemental analyser. Melting Points were measured using a Complab melting point instrument and are uncorrected.

**5-*O*-Desosaminyl-6-*O*-methylerythronolide A (1).** To 1 g (1.3 mmol) of clarithromycin was added 0.1 n aqueous hydrochloric acid (18 mL, containing 1.5 mL MeOH) and the mixture was stirred at room temperature for 4 h. The reaction mixture was adjusted to pH 10 with a 2 m NaOH solution, and was extracted with ethyl acetate. The organic layer was washed with brine solution. The organic layer was dried over Na_2_SO_4_ and evaporated in vacuo. The residue was crystallized from ethyl acetate to afford 700 mg (1.18 mmol, 89%) of **1** as colorless powder: mp 236–238 °C; IR (KBr) 1729, 1692 cm^−1^; ^1^H NMR (300 MHz, CDCl_3_): δ 2.66 (m, 1H), 1.26 (m, 3H), 3.50–3.58 (m, 1H), 2.12 (m, 1H), 1.13 (m, 3H), 3.69 (d, J = 1.8 Hz, 1H), 1.37 (s, 3H), 2.97 (s, 3H), 1.56 (dd, J_1_ = 1.8 Hz, J_2_ = 4.6 Hz, 1H), 1.90–1.96 (m, 1H), 2.58 (m, 1H), 1.13 (m, 3H), 3.01 (m, 1H), 1.13 (m, 3H), 3.86 (d, J = 1.2 Hz, H), 1.18 (s, 3H), 5.18 (dd, J_1_ = 1.9 Hz, J_2_ = 10.8, 1H), 1.49 (m, 1H), 1.90–1.96 (m, 1H), 0.84 (t, J = 7.6 Hz, 3H), 4.38 (d, J = 7.1 Hz, 1H), 3.24 (m, 1H), 2.47 (m, 1H), 2.25 (s, 6H), 1.20–1.28 (m, 1H), 1.67 (m, 1H), 3.50–3.58 (m, 1H), 1.26 (m, 3H); ^13^C NMR (75 MHz, CDCl_3_): 8.2, 10.4, 12.6, 15.2, 16.2, 17.7, 18.8, 21.2, 21.4, 28.0, 35.9, 37.5, 38.7, 40.2, 44.6, 45.5, 49.5, 65.7, 69.8, 70.3, 70.7, 74.2, 76.6, 78.0, 79.0, 88.5, 106.8, 175.0, 220.7; FABMS *m/z* 590 (M+H)^+^; Anal. Calcd for C_30_H_55_NO_10_: C, 61.10; H, 9.40; N, 2.37. Found: C, 60.87; H, 9.67; N, 2.09.

**3-*O*-{γ-[(Benzyloxycarbonyl)amino]butyryl}-5-*O*-desosaminyl-6-*O*-methylerythronolide A (2).** To a stirred solution of **1** (300 mg, 0.58 mmol) and γ‑[(benzyloxycarbonyl)amino]butyric acid (363.0 mg, 1.52 mmol, 3 equivalents) in 5 mL dichloromethane, was added 4-(dimethylamino)pyridine (185.4 mg, 1.52 mmol, 3 equivalents), followed by addition of 1,3-diisopropylurea (273.8 μL, 1.52 mmol, 3 equivalents) at 0 °C. The resulting mixture was stirred 30 min at 0 °C and then for 12 h at ambient temperature. 1,3-Diisopropylurea was removed through filtration, the reaction mixture was concentrated, dissolved in EtOAc, washed with a 5% aqueous sodium bicarbonate solution and brine. The organic layer was dried over Na_2_SO_4_ and concentrated under reduced pressure. The product was dissolved in 2.5 mL of MeOH and stirred for 6 h. The solvent was removed, and the residue was purified by column chromatography eluting with CHCl_3_/MeOH/25% NH_4_OH (97 : 3 : 0.1) to furnish 300 mg (0.37 mmol, 72%) of **2** as a creamy white amorphous solid: mp 122–123 °C; IR (KBr) 1737, 1698, 1663 cm^−1^; ^1^H NMR (300 MHz, CDCl_3_): δ 2.66 (m, 1H), 1.26 (m, 3H), 5.05 (d, J = 8.8 Hz, 1H), 2.12 (m, 1H), 1.13 (m, 3H), 3.69 (d, J = 1.8 Hz, 1H), 1.37 (s, 3H), 2.97 (s, 3H), 1.56 (m, 1H), 1.90–1.96 (m, 1H), 2.58 (m, 1H), 1.13 (m, 3H), 3.01 (m, 1H), 1.13 (m, 3H), 3.86 (d, J = 1.2 Hz, 1H), 1.18 (s, 3H), 5.18 (dd, J_1_ = 2.0 Hz, J_2_ = 10.7 Hz, 1H), 1.49 (m, 1H), 1.90–1.96 (m, 1H), 0.84 (t, J = 7.4 Hz, 3H), 4.01 (d, J = 7.6 Hz, 1H), 3.24 (m, 1H), 2.47 (m, 1H), 2.25 (s, 6H), 1.20–1.28 (m, 1H), 1.67 (m, 1H), 3.50–3.58 (m, 1H), 1.26 (m, 3H), 2.41–2.49 (t, 2H), 1.90–1.99 (m, 2H), 3.24–3.29 (m, 2H), 5.0 (s, 2H)*,* 7.15–7.35 (m, 5H); ^13^C NMR (75 MHz, CDCl_3_): 9.3, 10.8, 12.8, 15.6, 16.4, 18.3, 19.7, 21.2, 21.5, 26.1, 28.0, 33.5, 36.4, 37.5, 38.7, 40.2, 40.8, 43.2, 45.5, 49.5, 66.3, 66.7, 69.8, 70.0, 70.8, 74.2, 76.6, 78.3, 82.1, 88.5, 103.9, 128.5–128.9, 136.8, 156.8, 173.3, 174.1, 221.0; FABMS *m/z* 810 (M+H)^+^; Anal. Calcd for C_42_H_68_N_2_O_13_: C, 62.36; H, 8.47; N, 3.46. Found: C, 62.08; H, 8.43; N, 3.36.

**3-*O*-(γ-Aminobutyryl)-5-*O*-desosaminyl-6-*O*-methylerythronolide A (3).** Compound **2** (300 mg, 0.37 mmol) was dissolved in 20 ml of HPLC grade MeOH, to the above solution was added 100 mg of 10% Pd/C. Hydrogen was bubbled through this mixture under stirring for 30 min. The reaction mixture was filtered through celite, concentrated under reduced pressure to give **3**, 240 mg (0.35 mmol, 96%) as an amorphous colorless solid. ^1^H NMR (300 MHz, CDCl_3_): δ 2.66 (m, 1H), 1.26 (m, 3H), 5.05 (d, J = 9.1 Hz,1H), 2.12 (m, 1H), 1.13 (m, 3H), 3.69 (d, J = 1.8 Hz, 1H), 1.37 (s, 3H), 2.97 (s, 3H), 1.56 (dd, J_1_ = 1.8 Hz, J_2_ = 4.6 Hz, 1H), 1.90–1.96 (m, 1H), 2.58 (m, 1H), 1.13 (m, 3H), 3.01 (m, 1H), 1.13 (m, 3H), 3.86 (d, J = 1.2 Hz,1H), 1.18 (s, 3H), 5.18 (dd, 1H), 1.49 (m, 1H), 1.90–1.96 (m, 1H), 0.84 (t, J = 7.1 Hz, 3H), 4.01 (d, J = 7.6 Hz, 1H), 3.24 (m, 1H), 2.47 (m, 1H), 2.25 (s, 6H), 1.20–1.28 (m, 1H), 1.67 (m, 1H), 3.50–3.58 (m, 1H), 1.26 (m, 3H), 2.41–2.49 (t, 2H), 1.90–1.99 (m, 2H), 3.24–3.29 (m, 2H); ^13^C NMR (75 MHz, CDCl_3_): 9.3, 10.8, 12.8, 15.6, 16.4, 18.3, 19.7, 21.2, 21.5, 26.1, 28.0, 33.5, 36.4, 37.5, 38.7, 40.2, 40.8, 43.2, 45.5, 49.5, 66.7, 69.8, 70.0, 70.8, 74.2, 76.6, 78.3, 82.1, 88.5, 103.9, 173.3, 174.1, 221.0; FABMS *m/z* 676 (M+H)^+^.

**3-*O*-[γ-(4-Oxo-2-phenyl-thiazolidin-3-yl)butyryl]-5-*O*-desosaminyl-6-*O*-methylerythronolide A (4a) (method A).** To an ice cold solution of **3** (200 mg, 0.29 mmol) in dichloromethane was added benzaldehyde (122 μL, 1.16 mmol) and the resulting mixture was stirred for 5 min, followed by the addition of mercaptoacetic acid (113 μL, 1.74 mmol). After 5 min 1,3-dicyclohexylcarbodiimide (143.3 mg, 0.69 mmol) was added to the reaction mixture at 0 °C and the reaction mixture was stirred for additional 3 h at ambient temperature. 1,3-Dicyclohexylurea was removed by filtration and the filtrate was concentrated to dryness under reduced pressure. The residue was taken up in EtOAc, washed with 5% aqueous sodium bicarbonate and brine. The organic layer was dried over sodium sulfate and concentrated under reduced pressure to afford 240 mg of crude **4a** as a light yellow oil, which was purified over a silica column, eluting with CHCl_3_/MeOH/25% NH_4_OH (96 : 4 : 0.05) giving an oil, which was further precipitated by hexane, to give 118 mg of **4a** (0.14 mmol, 48% yield) as a fluffy white mass. mp 85–86 °C; IR (KBr) 1739, 1675, 1592 cm^−1^; ^1^H NMR (400 MHz, CDCl_3_): δ 0.84 (t, J = 7.07 Hz, 3H), 1.11 (m, 9H), 1.15 (s, 3H), 1.20–1.28 (m, 1H), 1.26 (m, 6H), 1.37 (s, 3H), 1.49 (m, 1H), 1.53–1.56 (dd, J_1_ = 1.8 Hz, J_2_ = 4.6 Hz, 1H), 1.72–1.82 (m, 1H), 1.90–1.96 (m, 2H), 1.90–1.99 (m, 2H), 2.17 (m, 1H), 2.47 (m, 1H), 2.32 (s, 6H), 2.52–2.56 (m, 1H), 2.66 (m, 1H), 2.77–2.82 (t, 2H), 3.01 (m, 1H), 3.03 (s, 3H), 3.23–3.26 (m, 1H), 3.24–3.29 (m, 2H), 3.50–3.58 (m, 1H), 3.69–3.73 (d, J = 1.8 Hz, 1H), 3.80 (s, 2H), 3.88–3.90 (d, J = 1.2 Hz, 1H), 4.01 (d, J = 7.6 Hz, 1H), 5.02–5.05 (d, J = 10.9 Hz, 1H), 5.15–5.17 (dd, J_1_ = 2.0 Hz, J_2_ = 10.8 Hz, 1H), 5.63–5.65 (d, J = 9.36 Hz, 1H)*,* 7.38–7.39 (d, J = 7.8 Hz, 2H), 7.30–7.31 (d, J = 5.7 Hz, 2H), 7.27 (s, 1H); ^13^C NMR (100 MHz, CDCl_3_): 8.6, 10.1, 12.1, 15.7, 17.6, 19.0, 20.8, 21.8, 28.3, 28.4, 31.3, 32.4, 35.8, 36.9, 38.3, 39.9, 42.1, 42.4, 45.0, 49.8, 63.2, 65.6, 69.1, 69.9, 73.8, 77.5, 77.7, 81.1, 88.5, 102.9, 126.6, 128.8, 171.1, 172.0, 173.3, 220.4; FABMS *m/z* 837 (M+H)^+^; Anal. Calcd for C_43_H_68_N_2_O_12_S·H_2_O: C, 60.40; H, 8.25; N, 3.28. Found: C, 60.40; H, 8.42; N, 3.25.

**3-*O*-{γ-[4-Oxo-2-(4-chlorophenyl)-thiazolidin-3-yl]butryl}-5-*O*-desosaminyl-6-*O*-methylerythronolide A (4b).** To an ice cold solution of **3** (200 mg, 0.29 mmol) in dichloromethane was added 4-chlorobenzaldehyde (165 mg, 1.16 mmol) and the resulting mixture was stirred for 5 min, followed by the addition of mercaptoacetic acid (113 μL, 1.74 mmol). After 5 min 1,3-dicyclohexylcarbodiimide (143.3 mg, 0.69 mmol) was added to the reaction mixture at 0 °C and the reaction mixture was stirred for additional 3 h at ambient temperature. 1,3-Dicyclohexylurea was removed by filtration and the filtrate was concentrated to dryness under reduced pressure. The residue was taken up in EtOAc, washed with aqueous sodium bicarbonate and brine, dried over sodium sulfate and concentrated under reduced pressure to deliver 200 mg of crude **4b** as a yellow oil, which was purified over a silica column, eluting with CHCl_3_/MeOH/25% NH_4_OH (97 : 3 : 0.05), the resulting oil was further precipitated by hexane, to give 103 mg of **4b** (0.11 mmol, 39% yield) as a fluffy white mass.

mp 102–105 °C; IR (KBr) 1736, 1677 cm^−1^; ^1^H NMR (400 MHz, CDCl_3_): δ 0.84 (t, J = 6.7 Hz, 3H), 1.13 (m, 9H), 1.18 (s, 3H), 1.20–1.28 (m, 1H), 1.26 (m, 6H), 1.37 (s, 3H), 1.49 (m, 1H), 1.56 (dd, J_1_ = 1.8 Hz, J_2_ = 4.6 Hz, 1H), 1.67 (m, 1H), 1.90–1.96 (m, 2H), 1.90–1.99 (m, 2H), 2.12 (m, 1H), 2.28 (s, 6H), 2.47 (m, 1H), 2.58 (m, 1H), 2.66 (m, 1H), 2.77–2.82 (t, 2H), 3.01 (m, 1H), 3.04 (s, 3H), 3.24 (m, 1H), 3.24–3.29 (m, 2H), 3.50–3.58 (m, 1H), 3.69 (d, J = 1.8 Hz, 1H), 3.81 (s, 2H), 3.86 (d, J = 1.2 Hz, 1H), 4.01 (d, J = 7.6 Hz, 1H), 5.03–5.06 (d, J = 10.9 Hz, 1H), 5.15–5.18 (dd, J_1_ = 2.1 Hz, J_2_ = 10.6 Hz, 1H), 5.61–5.63 (d, J = 10.5 Hz, 1H)*,*7.26 (d, J = 4.8 Hz, 2H), 7.35–7.37 (d, J = 8.2 Hz, 2H); ^13^C NMR (100 MHz, CDCl_3_): 8.9, 10.4, 12.5, 15.2, 16.0, 17.9, 19.3, 21.1, 22.0, 28.4, 29.6, 31.7, 32.7, 36.1, 37.2, 38.7, 40.2, 42.4, 42.7, 45.3, 50.1, 62.8, 66.0, 69.6, 70.3, 70.8, 74.1, 76.6, 78.1, 81.6, 90.4, 103.4, 128.4, 129.3, 135.1, 137.9, 171.3, 172.3, 173.6, 220.6; FABMS *m/z* 871 (M+H)^+^; Anal. Calcd for C_43_H_67_ClN_2_O_12_S: C, 59.26; H, 7.75; N, 3.21. Found: C, 58.91; H, 7.91; N, 2.93.

**3-*O*-{γ-[4-Oxo-2-(4-fluorophenyl)-thiazolidin-3-yl]butryl}-5-*O*-desosaminyl-6-*O*-methylerythronolide A (4c):** To an ice cold solution of **3** (200 mg, 0.29 mmol) in dichloromethane was added 4-fluorobenzaldehyde (125.0 μL, 1.16 mmol) and the resulting mixture was stirred for 5 min, followed by the addition of mercaptoacetic acid (113 μL, 1.74 mmol). After 5 min 1,3-dicyclohexylcarbodiimide (143.3 mg, 0.69 mmol) was added to the reaction mixture at 0 °C and the reaction mixture was stirred for additional 3 h at ambient temperature. 1,3-Dicyclohexylurea was removed by filtration and the filtrate was concentrated to dryness under reduced pressure. The residue was taken up in EtOAc, washed with sodium bicarbonate and brine, dried over sodium sulfate and concentrated under reduced pressure to deliver 200 mg of crude **4c** as a light yellow oil, which was purified over a silica column, eluting with CHCl_3_/MeOH/25% NH_4_OH (96 : 4 : 0.05), the resulting oil was further precipitated by hexane, to give 100 mg of **4c** (0.11 mmol, 41% yield) as a fluffy white mass. mp 103–105 °C; IR (KBr) 1736, 1677, 1603 cm^−1^; ^1^H NMR (400 MHz, CDCl_3_): δ 0.83 (t, J = 7.23 Hz, 3H), 1.12–1.13 (m, 3H), 1.13 (m, 9H), 1.19 (s, 3H), 1.20–1.29 (m, 1H), 1.29 (m, 6H), 1.27 (m, 3H), 1.52–1.55 (m, 1H), 1.75–1.82 (m, 1H), 1.90–1.96 (m, 1H), 1.50 (m, 1H) 1.90–1.96 (m, 1H), 1.90–1.99 (m, 2H), 2.12–2.22 (m, 1H), 2.26 (s, 6H), 2.33–2.36 (m, 1H), 2.51–2.56 (m, 1H), 2.53–2.55 (m, 2H), 2.75–2.85 (m, 1H), 2.99 (m, 1H), 3.03 (s, 3H), 3.24 (m, 1H), 3.24–3.29 (m, 2H), 3.59–3.65 (m, 1H), 3.69–3.71 (d, J = 1.8 Hz, 1H), 3.81 (S, 2H), 3.86–3.88 (d, J = 1.2 Hz, 1H), 3.90–3.92 (d, J = 7.6 Hz, 1H), 5.03–5.06 (d, J = 11.0 Hz, 1H), 5.15–5.18 (dd, J_1_ = 2.1 Hz, J_2_ = 10.5 Hz, 1H), 5.63–5.65 (d, J = 9.1 Hz, 1H), 7.06–7.10 (m, 2H), 7.29–7.33 (m, 2H); ^13^C NMR (100 MHz, CDCl_3_): 8.9, 10.4, 12.5, 15.2, 16.0, 17.9, 19.3, 21.1, 22.0, 28.4, 29.6, 31.7, 32.4, 36.1, 37.2, 38.7, 40.2, 42.4, 42.7, 45.3, 50.1, 62.3, 66.0, 69.6, 70.3, 70.8, 74.1, 76.6, 78.1, 81.6, 90.4, 103.4, 115.5, 128.6, 134.7, 163.9, 170.9, 172.3, 173.6, 220.6; FABMS *m/z* 855 (M+H)^+^; Anal. Calcd for C_43_H_67_FN_2_O_12_S: C, 60.40; H, 7.90; N, 3.28. Found: C, 60.55; H, 8.24; N, 3.16.

**3-*O*-{γ-[4-Oxo-2-(4-methoxyphenyl)-thiazolidin-3-yl]butryl}-5-*O*-desosaminyl-6-*O*-methylerythronolide A (4d)**: To an ice cold solution of **3** (200 mg, 0.29 mmol) in dichloromethane was added 4-methoxybenzaldehyde (143.5 μL, 1.16 mmol) and the resulting mixture was stirred for 5 min, followed by the addition of mercaptoacetic acid (113 μL, 1.74 mmol). After 5 min 1,3-dicyclohexylcarbodiimide (143.3 mg, 0.69 mmol) was added to the reaction mixture at 0 °C and the reaction mixture was stirred for additional 3 h at ambient temperature. 1,3-Dicyclohexylurea was removed by filtration and the filtrate was concentrated to dryness under reduced pressure. The residue was taken up in EtOAc, washed with sodium bicarbonate and brine, dried over sodium sulfate and concentrated under reduced pressure to deliver 200 mg of crude **4d** as a pale yellow oil, which was purified over a silica column, eluting with CHCl_3_/MeOH/25% NH_4_OH (96 : 4 : 0.05), the resulting oil was further precipitated by hexane, to give 98 mg of **4d** (0.11 mmol, 38% yield) as a fluffy white mass. mp 102–104 °C; IR (KBr) 1735, 1679 cm^−1^; ^1^H NMR (400 MHz, CDCl_3_): δ 2.66 (m, 1H), 1.26–1.28 (m, 3H), 5.02–5.05 (d, J = 10.9 Hz, 1H), 2.17 (m, 1H), 1.11 (m, 3H), 3.69–3.71 (d, J = 1.8 Hz, 1H), 1.37 (s, 3H), 3.03 (s, 3H), 1.53–1.56 (dd, J_1_ = 1.8 Hz, J_2_ = 4.6 Hz, 1H), 1.90–1.96 (m, 1H), 2.52–2.56 (m, 1H), 1.11 (m, 3H), 3.01 (m, 1H), 1.11 (m, 3H), 3.88–3.89 (d, J = 1.2 Hz, 1H), 1.17 (s, 3H), 5.15–5.18 (dd, J_1_ = 1.9 Hz, J_2_ = 10.9 Hz, 1H), 1.49 (m, 1H), 1.90–1.96 (m, 1H), 0.81–0.83 (t, J = 6.78 Hz, 3H), 4.01 (d, J = 7.6 Hz, 1H), 3.23–3.26 (m, 1H), 2.47 (m, 1H), 2.33 (s, 6H), 1.20–1.28 (m, 1H), 1.72–1.82 (m, 1H), 3.50–3.58 (m, 1H), 1.26 (m, 3H), 2.69–2.75 (t, 2H), 1.90–1.99 (m, 2H), 3.24–3.29 (m, 2H), 6.89–6.91 (d, J = 8.4 Hz, 2H), 7.24–7.27 (d, J = 9.7 Hz, 2H), 3.81 (s, 3H), 5.6 (d, J = 7.1 Hz, 1H), 3.78 (s, 2H); ^13^C NMR (100 MHz, CDCl_3_): 8.6, 10.1, 12.1, 15.7, 17.6, 19.0, 20.8, 22.0, 28.4, 29.3, 31.4, 32.6, 35.8, 36.9, 38.3, 39.9, 42.4, 45.0, 49.8, 55.9, 63.0, 65.7, 69.1, 69.2, 69.9, 73.8, 77.5, 77.7, 81.2, 90.4, 102.9, 114.1, 128.1, 130.5, 160.0, 171.0, 172.0, 173.3, 220.4; FABMS *m/z* 867 (M+H)^+^; Anal. Calcd for C_44_H_70_N_2_O_13_S·H_2_O: C, 59.71; H, 8.20; N, 3.16. Found: C, 60.02; H, 7.99; N, 2.99.

**3-*O*-{γ-[4-Oxo-2-(4-nitrophenyl)-thiazolidin-3-yl]butryl}-5-*O*-desosaminyl-6-*O*-methylerythronolide A (4e)**: To an ice cold solution of **3** (210 mg, 0.31 mmol) in dichloromethane was added 4-nitrobenzaldehyde (187.9 mg, 1.24 mmol) and the resulting mixture was stirred for 5 min, followed by the addition of mercaptoacetic acid (120 μL, 1.86 mmol). After 5 min 1,3-dicyclohexylcarbodiimide (153.7 mg, 0.74 mmol) was added to the reaction mixture at 0 °C and the reaction mixture was stirred for additional 3 h at ambient temperature. 1,3-Dicyclohexylurea was removed by filtration and the filtrate was concentrated to dryness under reduced pressure. The residue was taken up in EtOAc, washed with sodium bicarbonate and brine, dried over sodium sulfate and concentrated under reduced pressure to deliver 200 mg of crude **4e** as a yellow oil which was purified over a silica column, eluting with CHCl_3_/MeOH/25% NH_4_OH (97 : 3 : 0.05), the resulting oil was further precipitated by hexane, to give 120 mg of pure **4e** (0.13 mmol, 43% yield) as a fluffy creamy white mass. mp 107–110 °C; IR (KBr) 1736, 1681, 1605 cm^−1^; ^1^H NMR (400 MHz, CDCl_3_): δ 2.75–2.85 (m, 1H), 1.28 (m, 3H), 5.02–5.05 (d, J = 10.9 Hz, 1H), 2.12–2.22 (m, 1H), 1.12–1.13 (m, 3H), 3.69–3.72 (d, J = 1.8 Hz, 1H), 1.28 (s, 3H), 3.03 (s, 3H), 1.51–1.55 (dd, J_1_ = 1.8 Hz, J_2_ = 4.6 Hz, 1H), 1.90–1.96 (m, 1H), 2.51–2.56 (m, 1H), 1.13 (m, 3H), 2.99 (m, 1H), 1.13 (m, 3H), 3.85 (d, J = 1.2 Hz, 1H), 1.18 (s, 3H), 5.14–5.17 (dd, J_1_ = 2.0 Hz, J_2_ = 12.6 Hz, 1H), 1.51 (m, 1H), 1.90–1.96 (m, 1H), 0.83 (t, J = 7.1 Hz,3H), 3.95 (d, J = 7.6 Hz, 1H), 3.24 (m, 1H), 2.30–2.48 (m, 1H), 2.44 (s, 6H), 1.20–1.28 (m, 1H), 1.75–1.81 (m, 1H), 3.59–3.65 (m, 1H), 1.26 (m, 3H), 2.69–2.75 (t, 2H), 1.90–1.99 (m, 2H), 3.24–3.29 (m, 2H), 7.49–7.51 (d, J = 5.6 Hz, 2H), 8.25–8.28 (d, J = 8.3 Hz, 2H), 5.73–5.76 (d, J = 10.2 Hz, 1H), 3.80 (s, 2H); ^13^C NMR (100 MHz, CDCl_3_): 8.6, 10.1, 12.1, 15.7, 17.6, 19.0, 20.8, 22.0, 28.7, 29.3, 31.2, 32.2, 35.8, 36.9, 38.3, 39.4, 42.4, 44.9, 49.8, 62.0, 65.5, 68.6, 69.1, 69.6, 73.8, 77.0, 77.5, 81.3, 90.4, 102.8, 124.1, 124.2, 127.3, 127.4, 146.5, 147.9, 171.2, 172.0, 173.3, 220.4; FABMS *m/z* 883 (M+H)^+^; Anal. Calcd for C_43_H_67_N_3_O_14_S: C, 58.55; H, 7.66; N, 4.76. Found: C, 58.76; H, 7.85; N, 4.32.

**3-*O*-{γ-[4-Oxo-2-(4-quinolyl)-thiazolidin-3-yl]butryl}-5-*O*-desosaminyl-6-*O*-methylerythronolide A (4f)**: To an ice cold solution of **3** (200 mg, 0.29 mmol) in dichloromethane was added quinoline-4-carboxaldehyde (186.2 mg, 1.16 mmol) and the resulting mixture was stirred for 5 min, followed by the addition of mercaptoacetic acid (113 μL, 1.74 mmol). After 5 min 1,3-dicyclohexylcarbodiimide (143.3 mg, 0.69 mmol) was added to the reaction mixture at 0 °C and the reaction mixture was stirred for additional 4 h at ambient temperature. 1,3-Dicyclohexylurea was removed by filtration and the filtrate was concentrated to dryness under reduced pressure. The residue was taken up in EtOAc, washed with 5% aqueous sodium bicarbonate and brine, dried over sodium sulfate and concentrated under reduced pressure to deliver 200 mg of crude **4f** as an oil, which was purified over a silica column, eluting with CHCl_3_/MeOH/25% NH_4_OH (95 : 5 : 0.05), the resulting oil was further precipitated by hexane, to give 98 mg of pure **4f** (0.11 mmol, 38% yield) as a fluffy white mass. mp 95–97 °C; IR (KBr) 1735, 1680 cm^−1^; ^1^H NMR (400 MHz, CDCl_3_): δ 2.75–2.85 (m, 1H), 1.24 (m, 3H), 5.01–5.03 (d, J = 11.0 Hz, 1H), 2.12–2.15 (m, 1H), 1.12–1.13 (m, 3H), 3.59–3.59 (d, J = 1.8 Hz, 1H), 1.37 (s, 3H), 2.99 (s, 3H), 1.52–1.56 (dd, J_1_ = 1.8 Hz, J_2_ = 4.6 Hz, 1H), 1.90–1.96 (m, 1H), 2.51–2.56 (m, 1H), 1.13 (m, 3H), 2.99 (m, 1H), 1.13 (m, 3H), 3.64 (d, J = 1.3 Hz, 1H), 1.17 (s, 3H), 5.13–5.15 (dd, J_1_ = 1.9 Hz, J_2_ = 11.2 Hz, 1H), 1.49–1.52 (m, 1H), 1.90–1.96 (m, 1H), 0.83 (t, J = 6.7 Hz, 3H), 3.95 (d, J = 7.6 Hz, 1H), 3.24 (m, 1H), 2.30–2.48 (m, 1H), 2.25 (s, 6H), 1.20–1.28 (m, 1H), 1.69–1.80 (m, 1H), 3.59–3.65 (m, 1H), 1.24 (m, 3H), 2.69–2.75 (m, 2H), 2.12–2.15 (m, 2H), 3.22–3.24 (m, 2H), 7.13–7.14 (d, J = 3.8 Hz, 1H), 8.19–8.21 (d, J = 8.4 Hz, 1H), 7.65–7.68 (m, 1H), 7.78–7.82 (m, 1H), 7.86 (d, J = 4.77 Hz, 1H), 7.90–7.92 (d, J = 8.3 Hz, 1H), 6.38 (d, J = 10.2 Hz, 1H), 3.78 (s, 2H); ^13^C NMR (100 MHz, CDCl_3_): 8.7, 9.9, 15.0, 16.8, 17.4, 20.8, 22.0, 28.1, 28.5, 31.2, 32.0, 35.9, 37.0, 38.1, 40.0, 42.4, 43.3, 45.0, 49.9, 57.4, 65.7, 69.5, 70.1, 73.8, 77.5, 78.4, 81.7, 90.4, 103.6, 121.7, 124.6, 127.4, 128.7, 130.1, 144.1, 148.3, 149.2, 150.7, 171.3, 171.9, 173.2, 220.4; FABMS *m/z* 889 (M+H)^+^; Anal. Calcd for C_46_H_69_N_3_O_12_S·H_2_O: C, 60.97; H, 7.90; N, 4.64. Found: C, 61.28; H, 7.97; N, 4.38.

**Methyl γ-(4-oxo-2-aryl-thiazolidin-3-yl)butyrate (6a):** To the solution of **5** (2.340 g, 20 mmol) in THF was added DIEA (5.1 mL, 40 mmol) at rt and the resulting mixture was stirred for 2 min. To this reaction mixture was added benzaldehyde (4.02 mL, 40 mmol) and the resulting mixture was stirred for additional 5 min, followed by the addition of mercaptoacetic acid (4.8 mL, 60 mmol). It was then refluxed for 3 h. THF was evaporated in vacuo and the residue was taken up in EtOAc, washed with 5% aqueous citric acid solution, saturated sodium bicarbonate solution and brine respectively. The organic layer was dried over sodium sulfate and concentrated under reduced pressure to deliver 4.5 g of crude **6a** as yellow oil, which was purified over a silica column, eluting with EtOAc/Hexane (30 : 70), yielding 2.5 g of pure **6a** (8.9 mmol, 46% yield) as a yellow oil. ^1^H NMR (300 MHz, CDCl_3_): δ 3.81 (s, 3H) OCH_3_, 2.26 (m, 2H) CO-CH_2_, 1.72–1.83 (m, 2H) (CH_2_)_2_CH_2_, 3.65–3.89 (m, 2H) N-CH_2_, 7.28–7.41 (m, 5H), 5.65 (s, 1H) PhCH, 3.81 (s, 2H) SCH_2_; FABMS *m*/*z* 280 (M+H)^+^.

**γ-(4-Oxo-2-aryl-thiazolidin-3-yl)butyric acid (7a):** To a solution of **6a** (2 g, 7.14 mmol) in THF-MeOH-water (7 : 2 : 1) was added LiOH (321.4 mg, 7.8 mmol) and the reaction mixture was stirred for 3 h at rt. THF and MeOH were evaporated under reduced pressure and to the resulting residue was added aqueous citric acid solution till pH~3, and it was partitioned between EtOAc and 5% aqeuous brine solution. The organic layer was concentrated under reduced pressure to furnish 2.0 g of **7a** (7.6 mmol, 80%). ^1^H NMR (300 MHz, CDCl_3_): δ 2.26 (m, 2H) CO-CH_2_, 1.72–1.83 (m, 2H) (CH_2_)_2_CH_2_, 3.65–3.89 (m, 2H) N-CH_2_, 7.28–7.41 (m, 5H), 5.65 (s, 1H) PhCH, 3.81 (s, 2H) SCH_2_; ESMS *m*/*z* 266 (M+H)^+^.

**3-*O*-[γ-(4-Oxo-2-phenyl-thiazolidin-3-yl)butyryl]-5-*O*-desosaminyl-6-*O*-methylerythronolide A (4a) (method B).** To the solution of **1** (200 mg, 0.29 mmol) in DCM was added **7a** (215 mg, 1.03 mmol) and the resulting mixture was stirred for 5 min To this was added DMAP (125 mg, 1.024 mmol) followed by the addition of DIC (91 μL, 1.03 mmol) at 0 °C. The resulting mixture was stirred 30 min at 0 °C and then for 10 h at ambient temperature. 1,3-Diisopropylurea was removed through filtration, the reaction mixture was concentrated, dissolved in EtOAc, washed with 5% aqueous sodium bicarbonate and brine. The organic layer was dried over Na_2_SO_4_ and concentrated under reduced pressure. The product was dissolved in 2.5 mL of MeOH and stirred for 6 h. The solvent was removed, and the residue was purified by column chromatography eluting with CHCl_3_/MeOH/25% NH_4_OH (97 : 3 : 0.1) to furnish 15 mg (5.3%) of **4a** as light yellow amorphous. mp 84–85 °C; ^1^H NMR (400 MHz, CDCl_3_): δ 2.66 (m, 1H), 1.26–1.28 (m, 3H), 5.02–5.05 (d, J = 10.9 Hz, 1H), 2.17 (m, 1H), 1.11 (m, 3H), 3.69–3.73 (d, J = 1.8 Hz, 1H), 1.37 (s, 3H), 3.03 (s, 3H), 1.53–1.56 (dd, J_1_ = 1.8 Hz, J_2_ = 4.6 Hz, 1H), 1.90–1.96 (m, 1H), 2.52–2.56 (m, 1H), 1.11 (m, 3H), 3.01 (m, 1H), 1.11 (m, 3H), 3.88–3.90 (d, J = 1.2 Hz, 1H), 1.15 (s, 3H), 5.15–5.17 (dd, J_1_ = 2.0 Hz, J_2_ = 10.8 Hz, 1H), 1.49 (m, 1H), 1.90–1.96 (m, 1H), 0.83 (t, J = 7.06 Hz, 3H), 4.01 (d, J = 7.7 Hz, 1H), 3.23–3.25 (m, 1H), 2.47 (m, 1H), 2.26 (s, 6H), 1.20–1.28 (m, 1H), 1.72–1.82 (m, 1H), 3.50–3.58 (m, 1H), 1.26 (m, 3H), 2.77–2.82 (t, 2H), 1.90–1.99 (m, 2H), 3.24–3.29 (m, 2H), 7.38–7.39 (d, J = 7.8 Hz, 2H), 7.30–7.31 (d, J = 5.7 Hz, 2H), 7.27 (s, 1H), 5.63–5.65 (d, J = 9.36 Hz, 1H)*,* 3.80 (s, 2H); ^13^C NMR (100 MHz, CDCl_3_): 8.6, 10.1, 12.1, 15.7, 17.6, 19.0, 20.8, 21.8, 28.3, 28.4, 31.3, 32.4, 35.8, 36.9, 38.3, 39.9, 42.1, 42.4, 45.0, 49.8, 63.2, 65.6, 69.1, 69.9, 73.8, 77.5, 77.7, 81.1, 88.5, 102.9, 126.6, 128.8, 171.1, 172.0, 173.3, 220.4; FABMS *m*/*z* 837 (M+H)^+^; Anal. Calcd for C_43_H_68_N_2_O_12_S·H_2_O: C, 60.40; H, 8.25; N, 3.28. Found: C, 60.80; H, 8.42; N, 3.10.
