# Supplementary figures and images for: New acylides: synthesis of 3-O-[γ-(4-oxo-2-aryl-thiazolidin-3-yl)butyryl]erythromycin A derivatives
Source: Beilstein J Org Chem. 2008 May 13;4:14. doi: 10.3762/bjoc.4.14 (PMC2486485; doi:10.3762/bjoc.4.14)

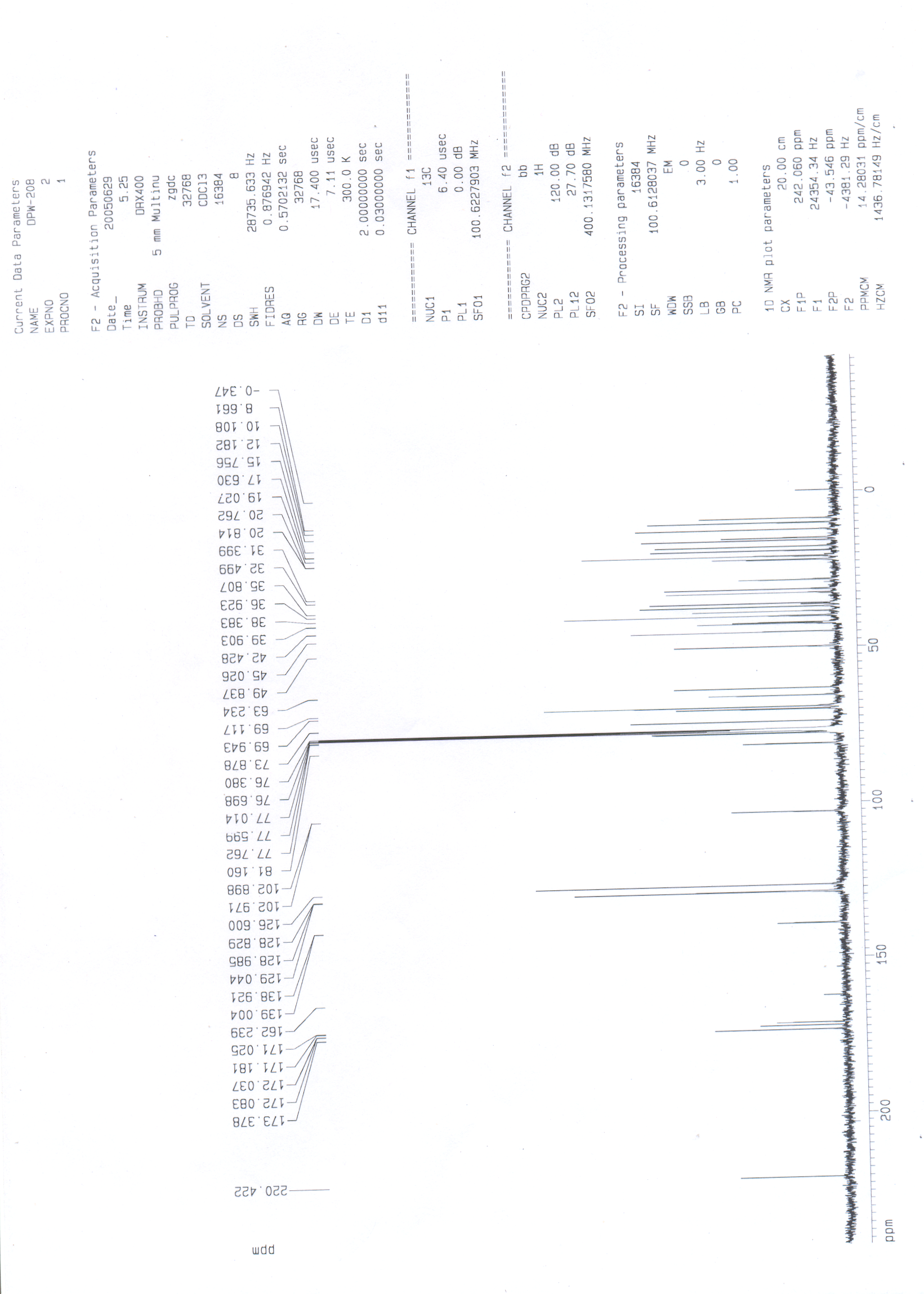


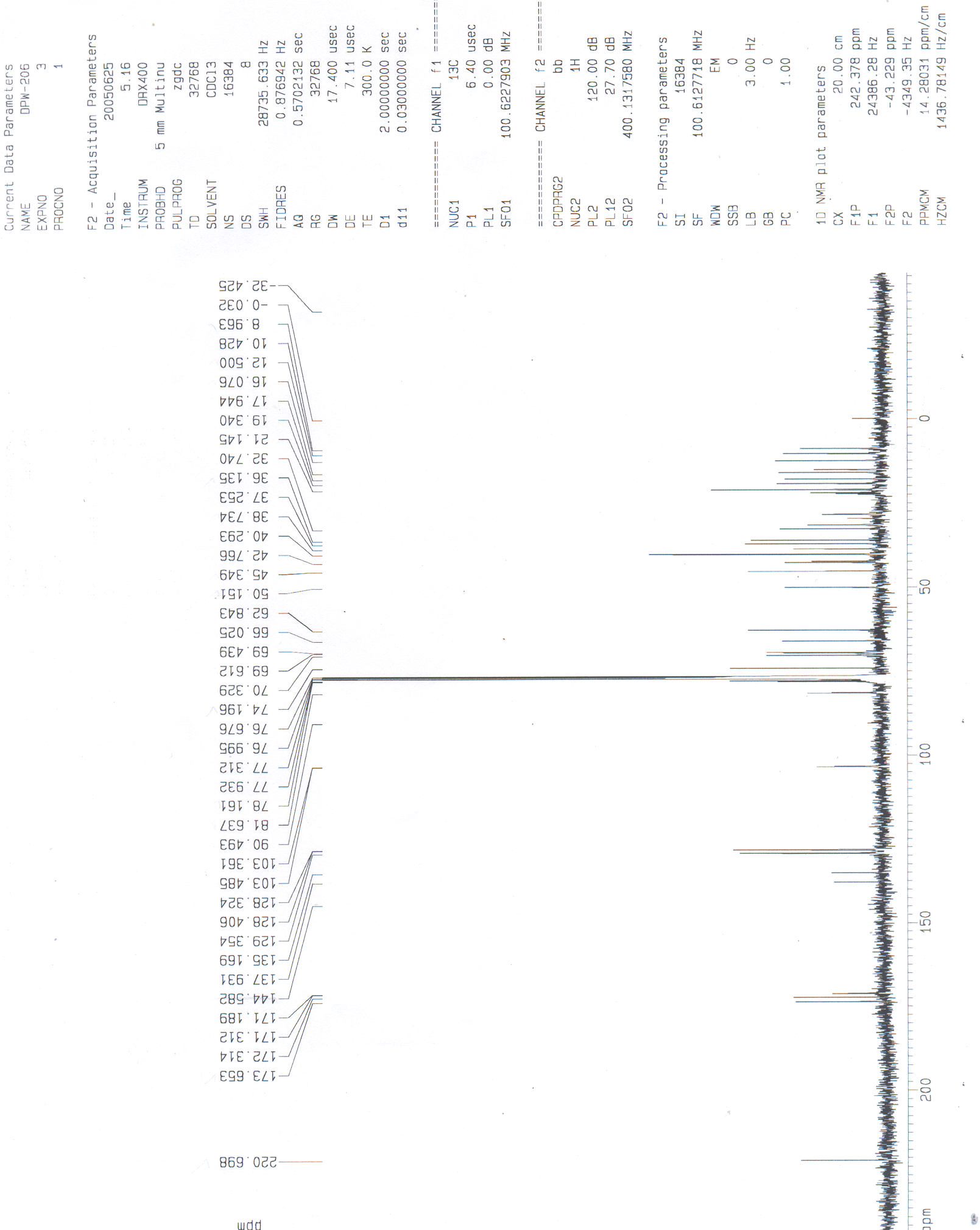


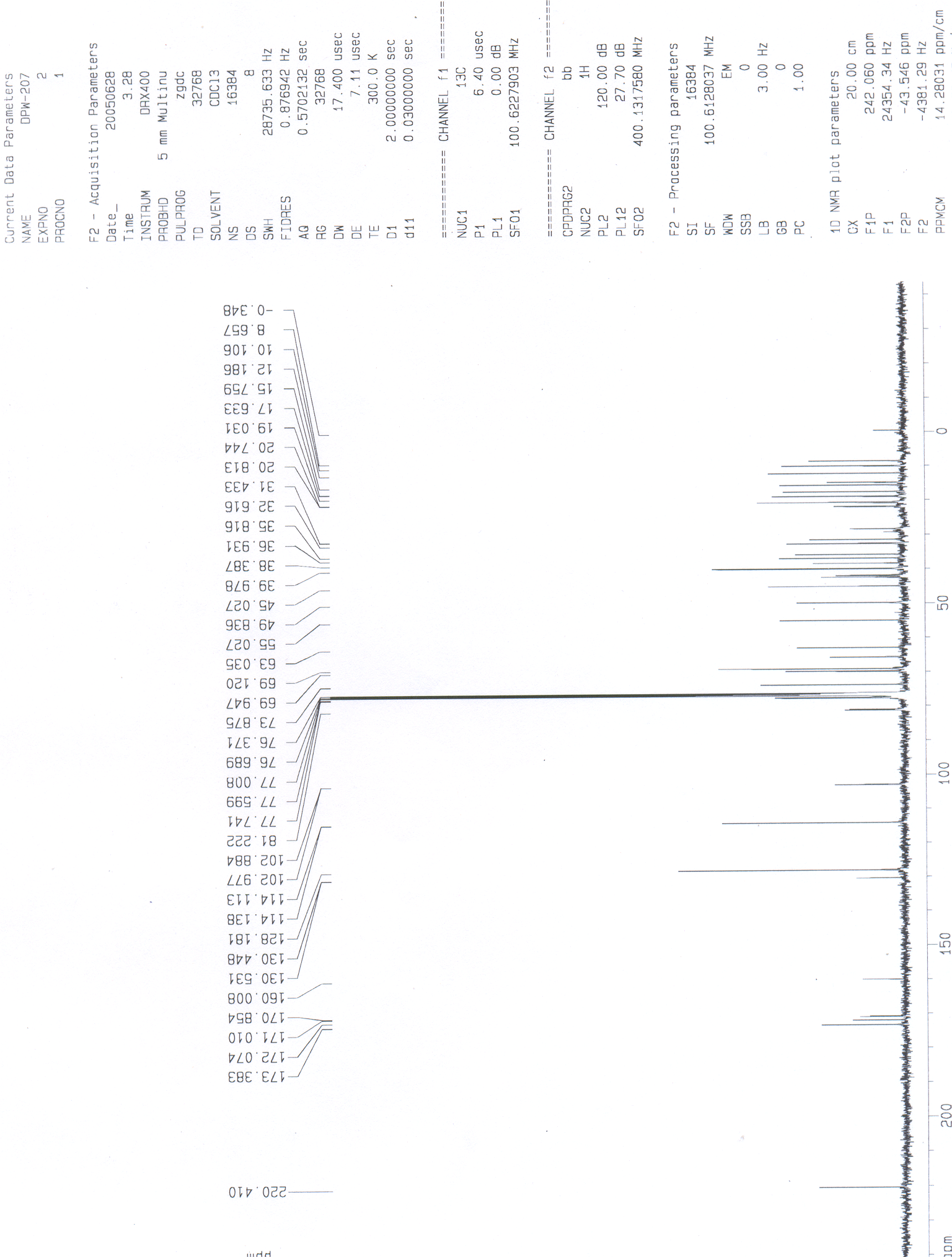


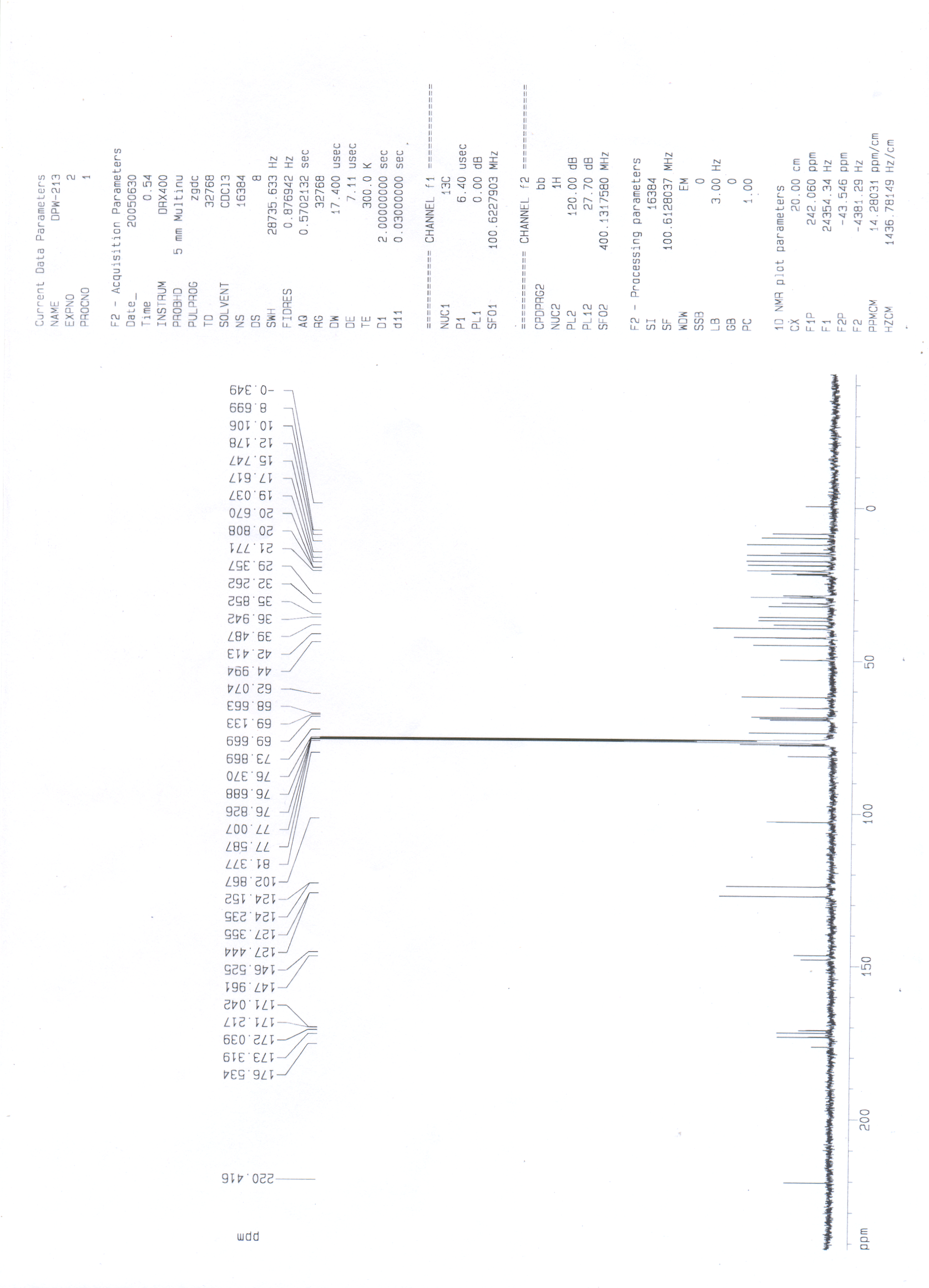

Supplement: File 2 — 1H NMR spectra of compounds 4a–d. [file Beilstein_J_Org_Chem-04-14-s002.doc]

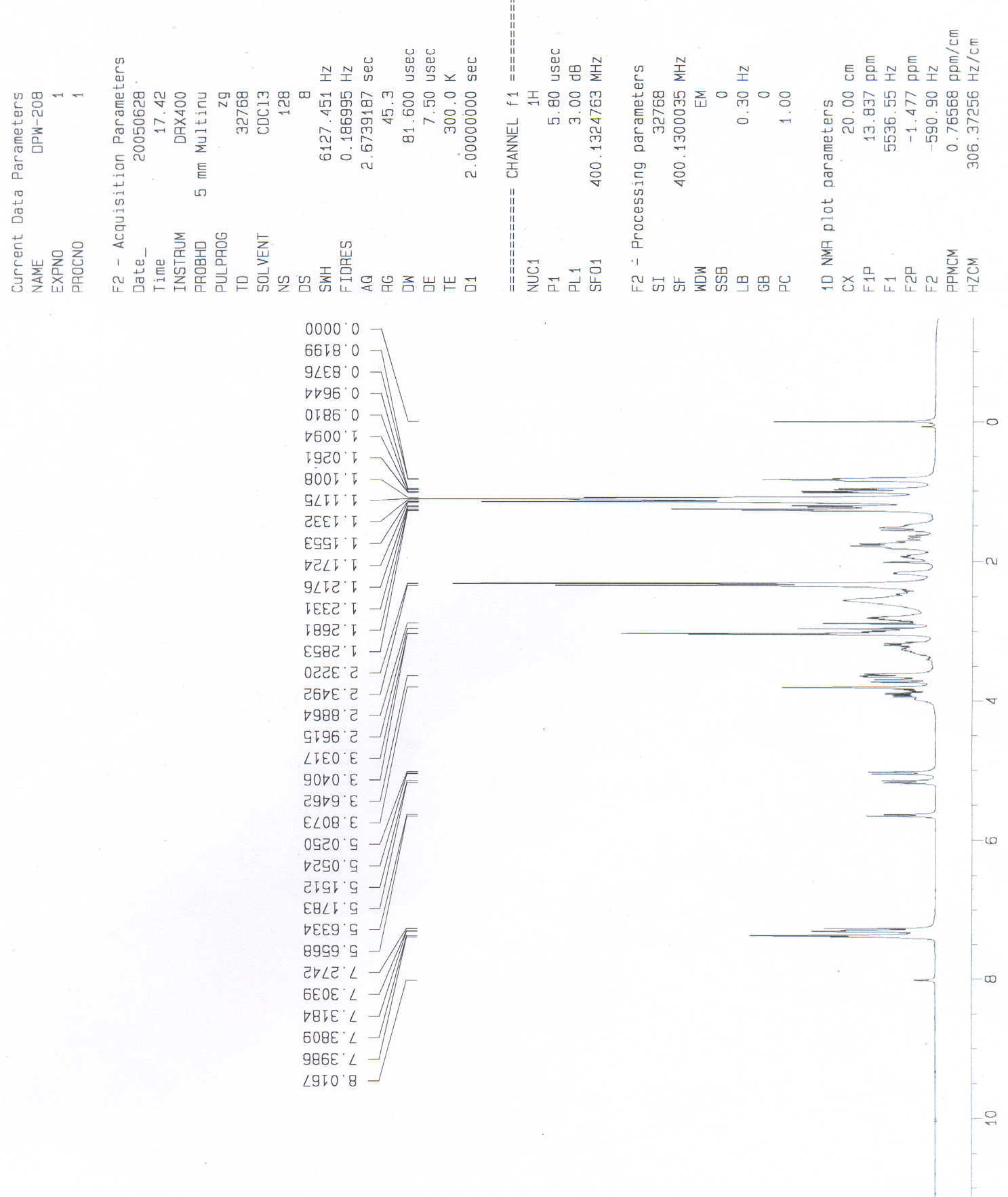


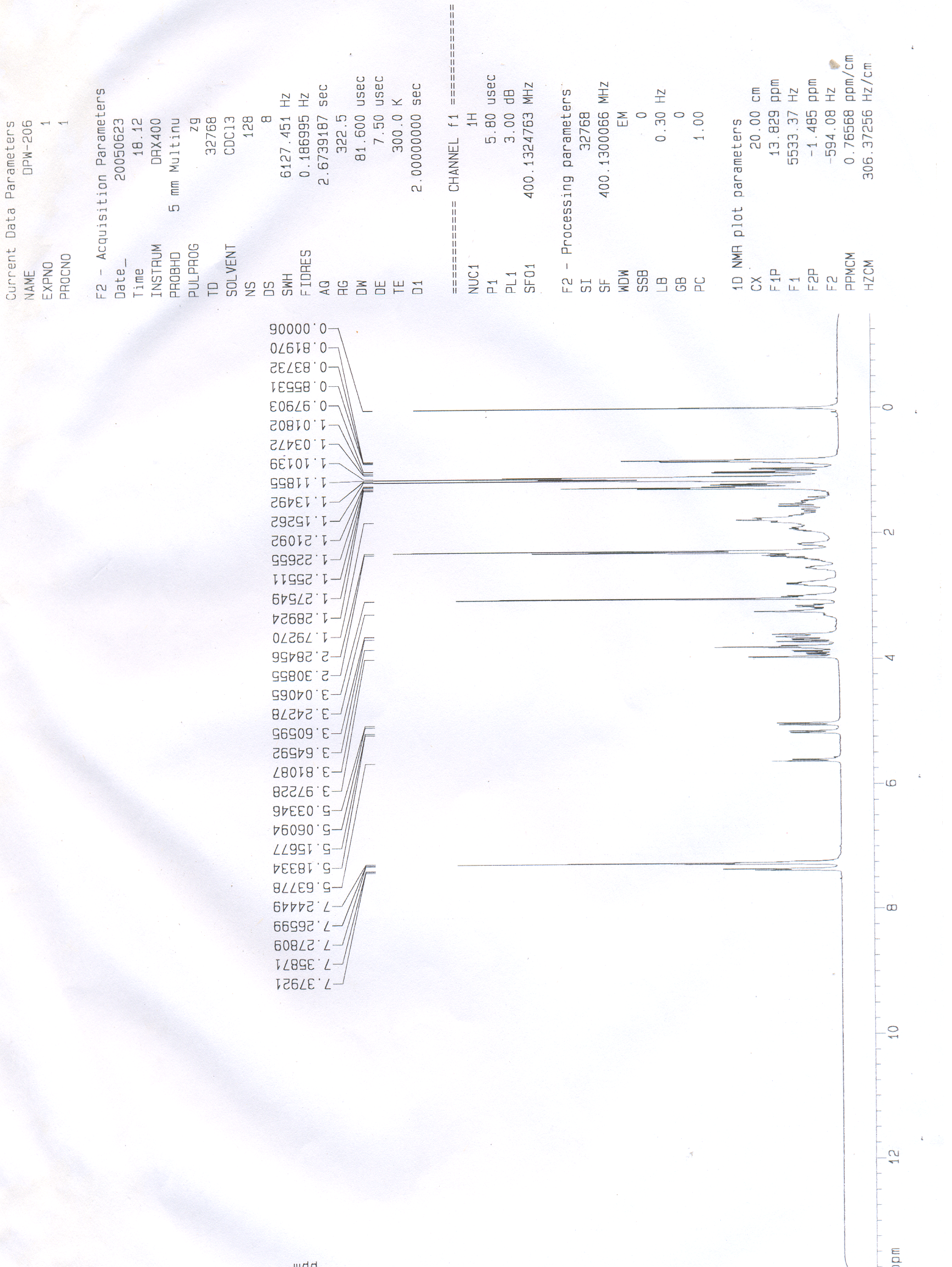


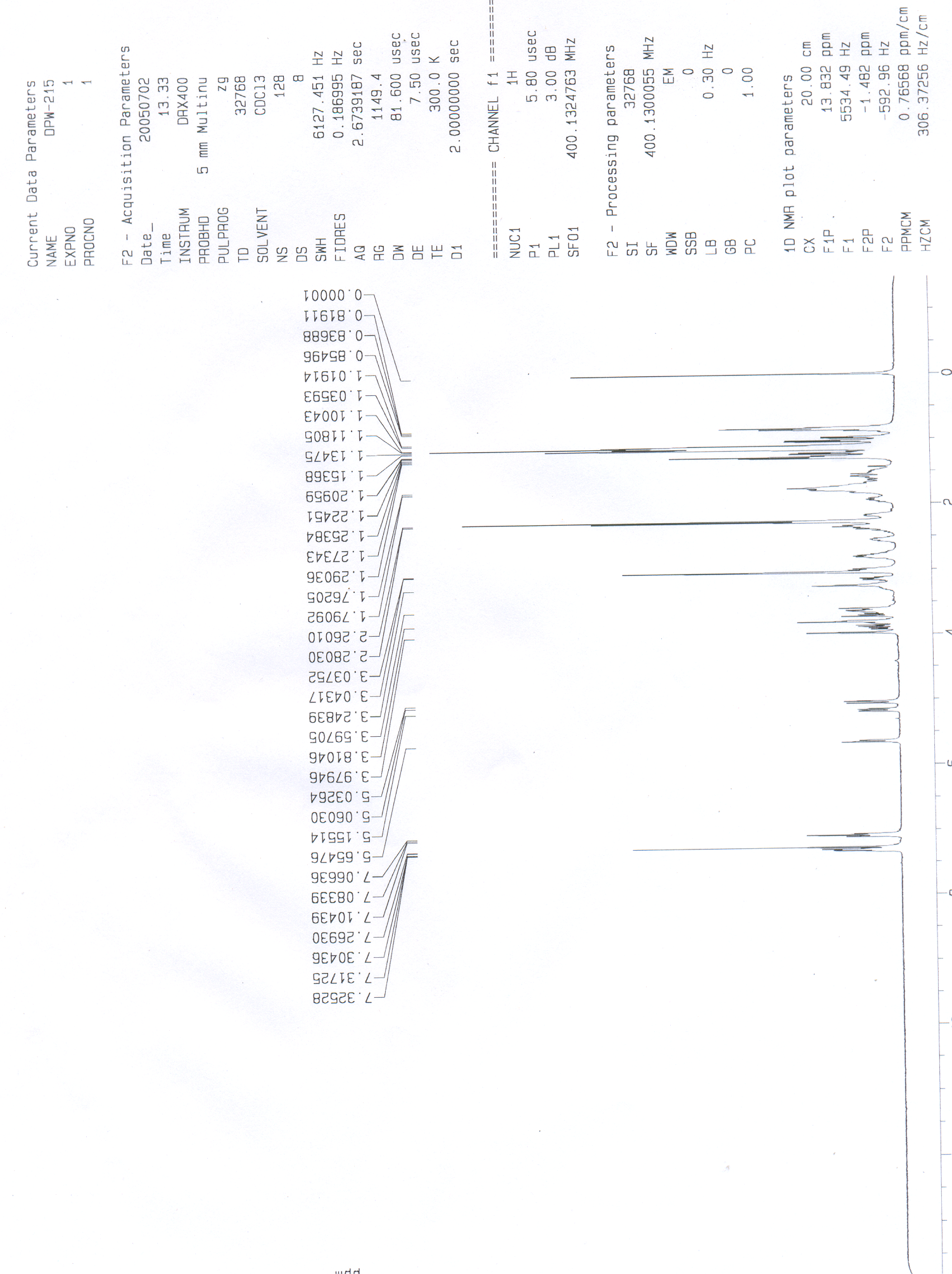


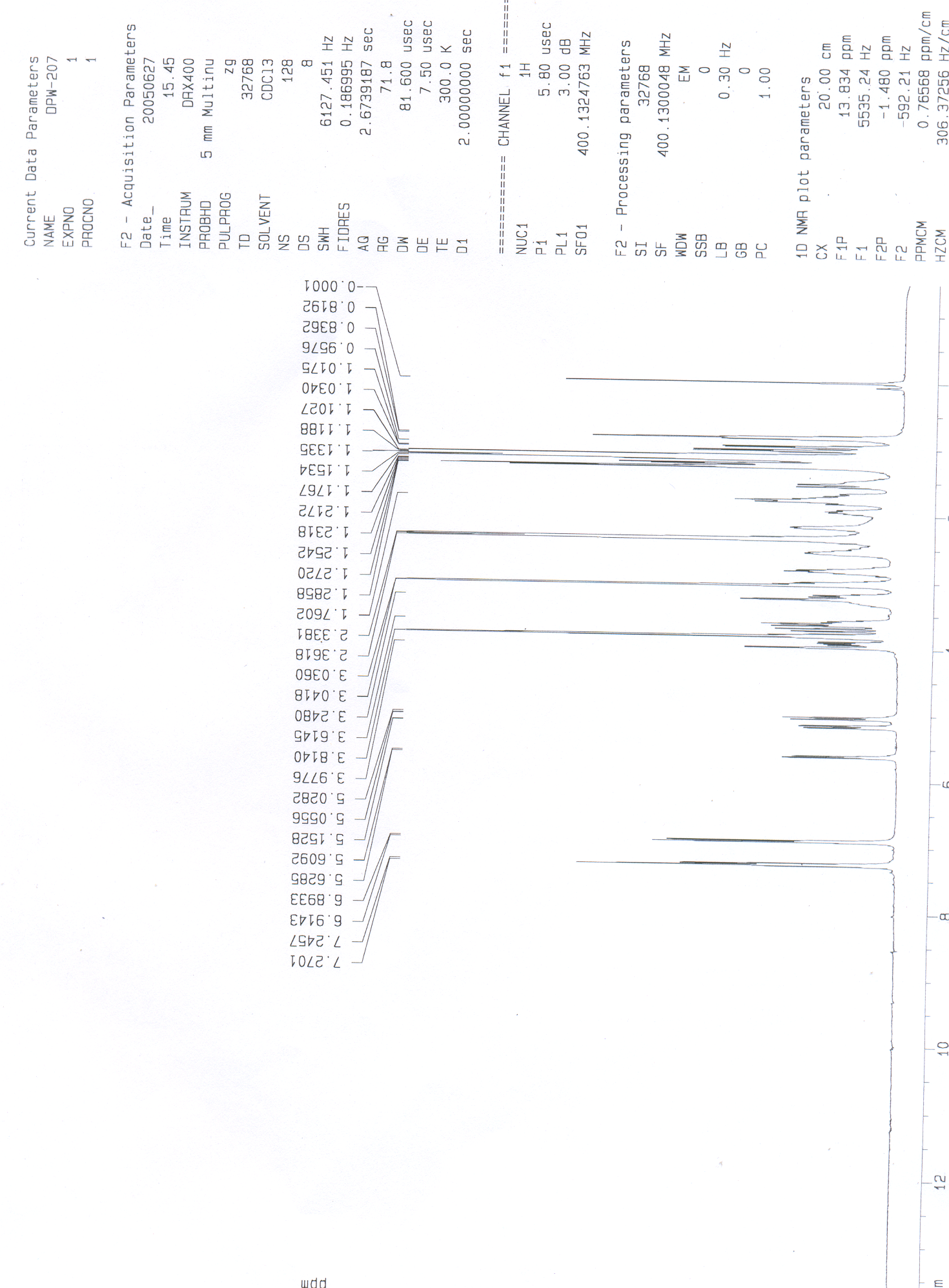

Supplement: File 3 — 13C NMR spectra of compounds 4a–b and 4d–e. [file Beilstein_J_Org_Chem-04-14-s003.doc]
